# Carbapenem or new β-lactam-β-lactamase inhibitors? An Italian survey supported by SITA, SIMIT and SIAARTI to identify the factors affecting empiric antimicrobial therapy choice in real-life clinical practice

**DOI:** 10.1007/s10096-024-04798-8

**Published:** 2024-03-08

**Authors:** Marta Colaneri, Camilla Genovese, Andrea Lombardi, Darcy Holmes, Alessandra Bandera, Andrea Gori

**Affiliations:** 1 Infectious Diseases and Immunopathology, Department of Clinical Sciences, Università di Milano, L. Sacco Hospital, Milan, Italy; 2 Centre for Multidisciplinary Research in Health Science (MACH), University of Milano, Milano, Italy; 3 Department of Pathophysiology and Transplantation, University of Milano, Milano, Italy; 4 Infectious Diseases Unit, Foundation IRCCS Ca’ Granda Ospedale Maggiore Policlinico, Milano, Italy

**Keywords:** Empirical antimicrobial therapies, Multidrug-resistant bacteria, Cephalosporin- resistant, Metallo-β-lactamase-producing enterobacterales rectal colonisation, MDROs, New β- lactams and β-lactamase inhibitors

## Abstract

While a tailored antibiotic treatment plan is often straightforward, what we often observe in daily clinical practice is a highly variable approach when defining empirical therapy. Specifically, a debate exists on preference to spare the new β-lactams and β-lactamase inhibitors (BL-BLIs) or to apply a carbapenem-sparing strategy first. To investigate, we designed a web survey aimed at investigating the variables considered relevant to empirically choosing one antibiotic over the other. Submitted to Italian infectious diseases and intensive care physicians through the support of Società Italiana di Malattie Infettive e Tropicali (SIMIT), Società Italiana di Terapia Antinfettiva (SITA) and Società Italiana Anestesia, Analgesia, Rianimazione e Terapia Intensiva (SIAARTI). We found that demographic characteristics were irrelevant when deciding for empirical therapy. Clinical and anamnestic data were most meaningful. Significantly considered were underlying comorbidities and previous exposure to antimicrobial treatments. History of third-generation cephalosporin-resistant, carbapenem-resistant and/or metallo-β-lactamase-producing Enterobacterales rectal colonisation and/or infection were considered the most relevant by most physicians. Unexpectedly, clinicians considered less the source of infection. These results prompt the need of straightforward methods to retrieve medical histories and the magnitude of rectal colonisation data, often not routinely obtained.

**Supplementary Information:**

The online version contains supplementary material available at 10.1007/s10096-024-04798-8.

## Background

Italy has currently reached hyper-endemic levels of multidrug-resistant microorganisms (MDROs) [1], with third-generation cephalosporin-resistant (3GCR) and carbapenem-resistant microorganisms leading the way [2]. While several guidelines support the choice of tailored treatment [3], there is a highly variable approach in defining the empiric one. In this regard, a specific debate exists on whether it’s preferable to spare the new β-lactams and β-lactamase inhibitors (BL-BLIs), such as ceftazidime-avibactam and ceftolozane/tazobactam or, conversely, apply a carbapenem-sparing strategy [4].

While it might seem reasonable to spare new molecules, potentially the last resort for MDROs, limiting the use of carbapenems might also be worthwhile, considering the growth of carbapenemase-producing bacteria.

Although some helpful risk scores have recently been proposed [5, 6], this doubt reflects different opinions among the referring infectious diseases (ID) and intensive care (IC) Italian centres.

Hence, it becomes relevant to know exactly which variables are considered by ID and IC clinicians to choose the empiric therapy. To investigate this intriguing issue, we designed a web survey directed toward Italian ID and IC physicians involved in prescription of antibiotics for infections due to MDRO support through Società Italiana di Malattie Infettive e Tropicali (SIMIT), Società Italiana di Terapia Antinfettiva (SITA) and Società Italiana Anestesia, Analgesia, Rianimazione e Terapia Intensiva (SIAARTI).

## Materials and methods

### Study setting

A web survey developed by ID teams of Ospedale Luigi Sacco and Ospedale Maggiore Policlinico, Milan, Italy, between November and December 2022, was hosted in an internet service (WebGoogle Forms), and diffused on January 5th, 2023, by email. The link was available on SIMIT’s website from March 27th, 2023. Answers were collected from March 1st to May 21st 2023. No limits were placed on the number of responses. Our goal was to collect at least one answer from each Italian administrative region, see Figure S1 in the Supplementary Materials 1.

### Survey structure

The survey composed 31 questions in 3 sections. The first section collected physicians’ data and centres’ general characteristics. Participants were then asked whether they would empirically consider starting a new BL-BLI, acting as a segue to the details of what would specifically prompt this choice. In fact, the second section explored all specific variables that might be relevant in starting an empirical antimicrobial treatment with novel BL-BLIs. Here, clinicians were required to assign a numerical weight referring to the grade of significance of each variable (0 to 3; not relevant to extremely relevant). Finally, the third section was composed of open-ended questions regarding considerations from the clinicians when choosing empiric therapy, facilitating a more thorough understanding of the physicians’ perspectives.

Access to the full questionnaire is available in the *Supplementary Materials 2*.

### Statistical analyses

Continuous variables are presented as medians and interquartile ranges, and categorical variables as frequencies and percentages. Analyses and graphical illustrations were performed with free software R version 3.5.1.

## Results

Regarding the first section, 171 physicians answered the web survey (Table 1).

**Table 1:** Participants’ characteristics

Variable			n (%)	
Gender		Male		84 (49.1)
Age [years, mean (SD)]	< 30 years			17 (9.9)
	30–40 years			65 (38)
	40–50 years			39 (22.8)
	> 50 years			50 (29.2)
Current position	Trainee			26 (15.2)
	Specialist			145 (84.8)
Specialty	Infectious Diseases			36 (21.1)
	Intensive care			135 (78.9)
Years of experience	< 5 years			70 (40.9)
	5–10 years			20 (11.7)
	> 10 years			81 (47.4)
Type of Hospital	University			111 (64.9)
	Non-university			60 (35.1)
Administrative regions of the Italian Republic	Abruzzo			3 (1.8)
	Basilicata			1 (0.6)
	Calabria			4 (2.3)
	Campania			11 (6.4)
	Emilia Romagna			11 (6.4)
	Friuli Venezia Giulia			3 (1.8)
	Lazio			19 (11.1)
	Liguria			5 (2.9)
	Lombardia			52 (30.4)
	Marche			5 (2.9)
	Molise			1 (0.6)
	Piemonte			5 (2.9)
	Puglia			8 (4.7)
	Sardegna			5 (2.9)
	Sicilia			13 (7.6)
	Toscana			12 (7.0)
	Trentino			1 (0.6)
	Umbria			1 (0.6)
	Val d’Aosta			2 (1.2)
	Veneto			9 (5.3)

Overall, 49.1% were male and 38% were aged between 30 and 40 years. Approximately 84.8% were specialised in either ID or IC by the collection time, while 15.2% were trainees.

The ID specialists represented most of the physicians responding (78.9%), and more than half (64.6%) worked in university hospitals.

**Fig. 1:**
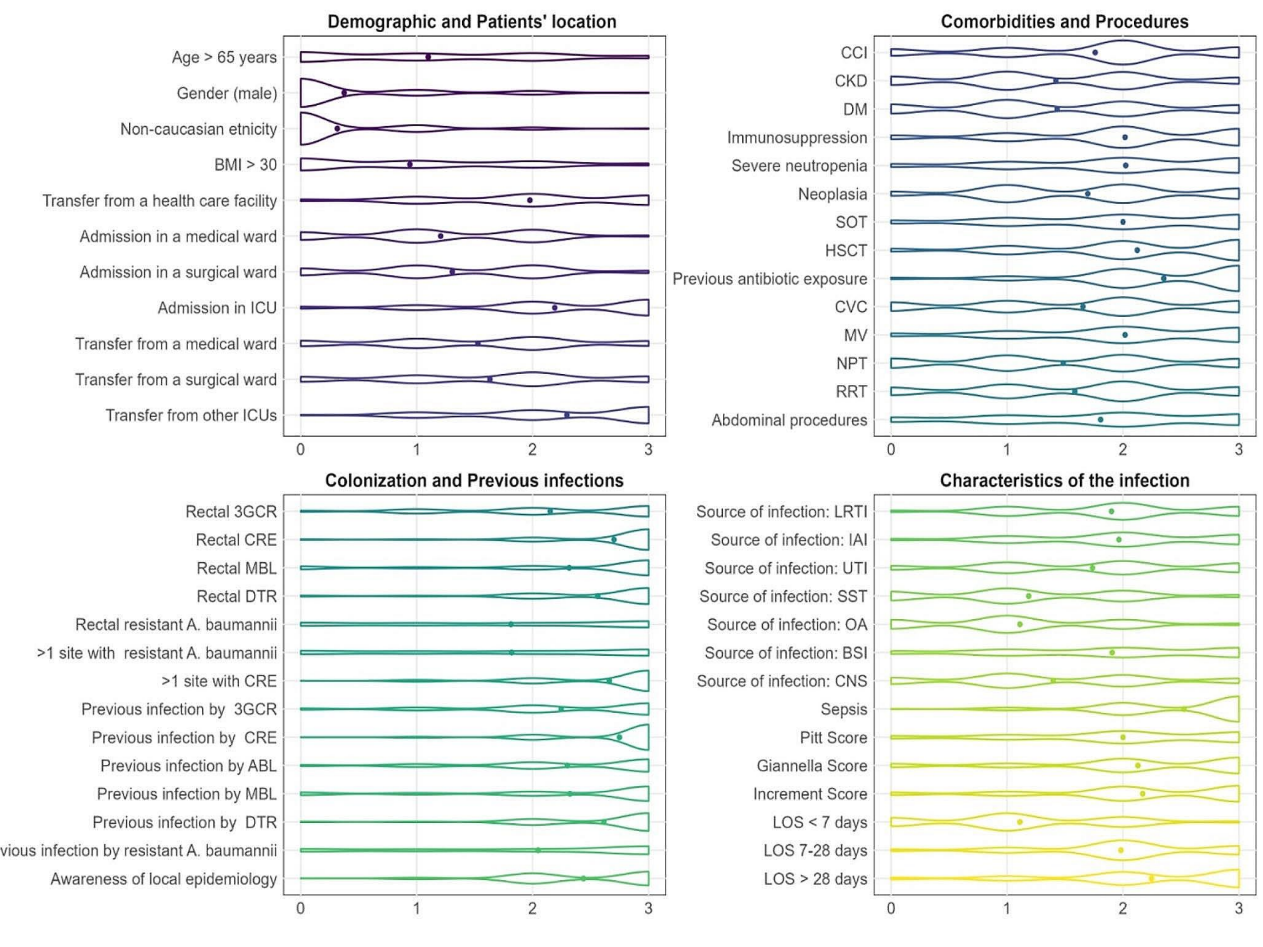
Percentage of agreement on the weight (from 0 to 3) assigned to the considered variables, subdivided into demographic and patients clinical, microbiological (colonisation and previous infections) and current infection characteristics by the overall cohort of physicians (*n* = 171). BMI, body mass index; CCI, Charlson’s comorbidity index; CKD, chronic kidney disease; DM, diabetes mellitus; SOT, solid organ transplantation; HSCT, haematopoietic stem cell transplantation; CVC central venous catheter; MV, mechanical ventilation; NPT, parenteral nutrition; RRT, renal replacement therapy; 3GCR, third-generation cephalosporin-resistant microorganism; CRE, carbapenem-resistant Enterobacteriaceae; MBL, metallo-β-lactamase-producing microorganism; DTR, difficult-to-treat resistant microorganism; ABL, AmpC β-lactamase-producing Enterobacteriaceae; LRTI, lower respiratory tract infection; IAI, intra-abdominal infection; UTI, urinary tract infection; SST, skin and soft tissue infection; OA, osteoarticular infection; BSI, primary bacteraemia; CNS, central nervous system infection; LOS, length of stay. The questions involving the variables *admission in a medical ward*, *admission in a surgical ward* and *admission in ICU* were dedicated only to the infectious disease specialists. *Immunosuppression* was defined as follows: patients on steroid therapy (> 20 mg of prednisone daily for > 15 days), or other immunosuppressive treatments, haematological malignancy, HIV infection, SOT and HSCT. *Severe neutropenia* was defined as an absolute neutrophil count of < 500/mmc. The variable *sepsis* was defined as the presence of sepsis or sepsis shock defined by Sepsis-3 [9]. By *rectal 3GCR, rectal CRE, rectal MBL, rectal DTR* and *rectal resistant A. baumannii* we mean rectal colonisation detected by rectal swab by the indicated microorganisms. By *> 1 site with* we mean patients who are colonised in more than one site other than rectal colonisation by the indicated microorganisms. By previous infection we mean a known previous infection with the specific pathogens in the previous 90 days. By awareness of local epidemiology we mean awareness of the resistant mechanisms of epidemiology of the participants’ centres

Regarding the preliminary question concerning empirical consideration of starting a new BL-BLI, over half (57.3%) answered yes. The second section of the web survey are shown in Fig. 1, which summarises the grade of significance that clinicians attributed to each variable for deciding to start an empiric therapy with a new BL-BLI.

Patients’ demographic characteristics were rated non-relevant by most physicians, with an assigned score of 0 by 62%, 74.3%, and 76.6% of them, respectively.

Significantly, when examining patients’ location, roughly half of participants deemed the ICU data to be of high-medium relevance, scoring 2 for patient admission to or transfer from an ICU.

Considering comorbidities and procedures, the two variables jointly considered the main relevant were solid organ transplant recipients, with 35.7% of the clinicians attributing a value of 3, and hematopoietic stem-cell transplantation, with 40.9% of participants scoring 3.

Half of the physicians considered also previous exposure to antimicrobial therapies in ≤ 90 days as extremely important when deciding whether to start a new BL-BLI empirically.

Third, from a microbiological point of view, rectal colonisation by 3GCR, carbapenem-resistant (CRE) and/or metallo-β-lactamase (MBL)-producing Enterobacterales was considered highly relevant, with a value of 3 attributed by 45.8%, 78.9% and 64.3% of the participants, respectively. Similar results were obtained regarding previous infection in ≤ 90 days by CRE and MBL-producing Enterobacterales.

The microbiological variables and their associated values are reported in Supplementary Materials 1, Table S1.

When asked about the type of infection, most physicians attributed to primary bacteraemia, lower respiratory tract infections, and intra-abdominal infections significantly higher scores compared to osteoarticular, skin and soft-tissue and urinary tract infections. Moreover, severity of disease was perceived as meaningful as the presence of sepsis, according to the Sepsis-3 criteria [7], was scored 3 by 62% of physicians. The Giannella [5] and the Increment [6] scores were almost unanimously scored a weight of 2.

Regarding the length of stay (LOS), burden increases proportionally to its duration. Specifically, while a LOS of 7 days was evaluated as relevant (score of 3) in starting a new BL-BLI by 3.5% of physicians, this percentage rose to 24% for a LOS of 7–28 days and 47.4% for a LOS more than 28 days.

Finally, regarding the third section of the web survey, where physicians were free to specify variables they consider relevant when choosing empiric therapies, only 19% (33/171) answered. Among them, variables included the availability of fast microbiological techniques, patient’s prognosis, presence of wounds or burns, and the previous treatment failure with a new BL-BLI.

Participants were also explicitly asked why it would be more appropriate to spare either carbapenems or new BL-BLIs when empirically chosen.

Seventy clinicians (40.9%) and 63 (36.8%) thought it was reasonable to spare carbapenems and new BL-BLIs, respectively. In both cases, the choice was related to the concern of producing and selecting resistant strains. Particularly, some clinicians defined BL-BLIs as last-resource molecules, only to be used after testing or considering all possible alternatives.

## Discussion

A total of 171 physicians responded to our web survey, mostly ID specialists, to whom the intra-hospital prescription of empiric antimicrobial is usually reserved [8], working in academic hospitals, where physicians are theoretically more familiar with evidence-based medicine. Furthermore, we achieved a comprehensive snapshot of the national landscape, with at least one answer from each Italian administrative region. According to our main findings, when examining patients’ demographic characteristics and location, while the former was negligible in deciding whether to start empiric treatment with the new BL-BLIs, the admission in and transfer from the ICUs played a significant role.

Differently, the patient’s clinical and anamnestic data were the most meaningful by the majority of physicians. Specifically, all-cause immunodepression, previous exposure to antimicrobial treatment, and history of 3GCR, CRE and MBL-producing Enterobacterales rectal colonisation or infection. Unexpectedly, the source of infection, despite still carrying a burden of significance, was less highly quoted.

The most recent knowledge broadly supports our results. Demographic characteristics are also not commonly considered in the most widely applied risk scores for infection and mortality by MDROs [6], which may well justify the prescription of novel antibiotics. Similarly, since ICUs are regarded worldwide as the epicenter of development and dissemination of MDROs [9], without exceptions for the specific Italian scenario [10], it is not astonishing that coming from or being admitted to ICUs was at least deemed worthy of consideration in empirically choosing a novel BL-BLIs.

Regarding the underlying comorbidities, although the exact role played by the immune system in regulating antimicrobial resistance is scarcely defined [11], greater rates of infections with MDROs are generally observed in immunocompromised hosts [12]. Potential explanations may lie in the higher use of antibiotics in this specific population, which notoriously advances the emergence of MDROs [13], the frequent access to healthcare facilities, and the lack of additional selective pressure imposed by immunocompetence against pathogen replication, which enhances their survival potential sufficiently to increase resistance [14]. With this in mind, it was not unexpected to find all-cause immunosuppression, as well as the previous use of antibiotics, as critical conditions to select an empiric antibiotic treatment based on novel BL-BLIs, which are helpful against infections probably due to MDROs.

It is appropriate to stress one point that we identified as unquestionably crucial for most of the interviewed clinicians in selecting an empiric antibiotic therapy with the novel BL-BLIs, namely the awareness of colonisation and previous infection with MDROs. It is currently well-known that colonisation with 3GCR, CRE and MBL-producing Enterobacterales significantly increases the risk of multidrug-resistant infections [15, 16] and that readmissions among patients with MDROs infections frequently happen for the same reason [17]. There are conflicting views on the benefits of mass rectal screening. While the German and Dutch health authorities [18, 19] suggest an active screening, the US guidelines do not [20], with some authors claiming that the identification MDROs carriers might increase the risk of broad-spectrum antibiotic overuse, eventually leading to negative consequences for both individuals and society [21].

The utmost significance of this data in our web survey prompts us to strongly believe that it would be valuable to reconsider these thoughts, at least to screen those patients at high risk for MDROs infections.

As we are aware that scores are renowned for reducing uncertainty, prompt missed diagnoses and increasing efficiency [22], we expected both the Giannella [5] and the Increment [6] scores to achieve a significantly high ranking. However, the answers confirmed that *real-life* clinical practice is far more complex than we can outline and frequently departs from established schemes.

Our survey was conducted in a context where the lack of clear guidelines for empirical therapy, especially with the new BL-BLIs, has made formulating precise guidelines a challenging task. The complexity of the issue is further highlighted by Bassetti et al. in a recent narrative review, emphasizing that novel agents should align with antimicrobial stewardship principles to combat resistance without excessive restrictions, which might hinder access to crucial treatments for severe difficult-to-treat infections [23].

This underscores the importance of our study in capturing nuances in clinical practice and contributing significantly to the ongoing discussion on empirical therapy with new BL-BLIs.

Finally, despite providing an overview of ID specialists’ point of view on empiric antibiotic therapy, few limitations should be acknowledged. First, the survey was conducted among physicians who voluntarily participated, which may introduce sampling bias, as those who chose to respond might have specific interests or experiences that differ from those who did not participate. Secondly, the data collected relies on self-reporting by physicians, which may be subject to recall bias, as physicians may not accurately remember their decision-making process or may provide socially desirable responses. Thirdly, the majority of respondents were ID specialists, which may limit the generalizability of your findings to other medical specialties. Finally, our sample size is small and circumscribed to Italy, which is nevertheless sadly known to be affected by one of the highest rates of MDROs worldwide.

Despite these limitations, our findings indicate how no clear guidance is available concerning the choice of an empiric antibiotic therapy in high MDROs endemic settings, like Italy. Stronger evidence is therefore required to provide answers to this complex issue.

For all these reasons, we believe that our results concretely support who deals every day with the choice of empiric antimicrobic therapy, especially in the long-lasting dilemma of picking what is optimal for patients, whilst simultaneously avoiding any harm in terms of the emergence of antimicrobial resistance.

## Conclusion

In conclusion, our survey highlights the pivotal role of patients’ clinical history and colonisation/previous infection status, together with the awareness of local epidemiology of multidrug-resistant organisms in physicians’ decisions to initiate empiric antimicrobial therapy with novel BL-BLIs. While acknowledging limitations such as sampling bias and self-reporting, our findings underscore the complexity of *real-life* decision-making, emphasizing the need for continued research to refine and enhance guidelines for optimizing antibiotic use in clinical practice.

### Electronic supplementary material

Below is the link to the electronic supplementary material.

Supplementary Material 1

Supplementary Material 2

## Data Availability

Not applicable.
