# Supplementary material for: Carbapenem or new β-lactam-β-lactamase inhibitors? An Italian survey supported by SITA, SIMIT and SIAARTI to identify the factors affecting empiric antimicrobial therapy choice in real-life clinical practice
Source: Eur J Clin Microbiol Infect Dis. 2024 Mar 8;43(5):1017–23. doi: 10.1007/s10096-024-04798-8 (PMC11108948; doi:10.1007/s10096-024-04798-8)
Supplement: Supplementary file 1 — Supplementary Material 1 [file 10096_2024_4798_MOESM1_ESM.docx]

**Supplementary Materials:**

**Figure S1:** Geographic distribution of the physicians who answered the survey. The size of the dots is directly proportional to the number of answers received by each administrative region of the Italian Republic.


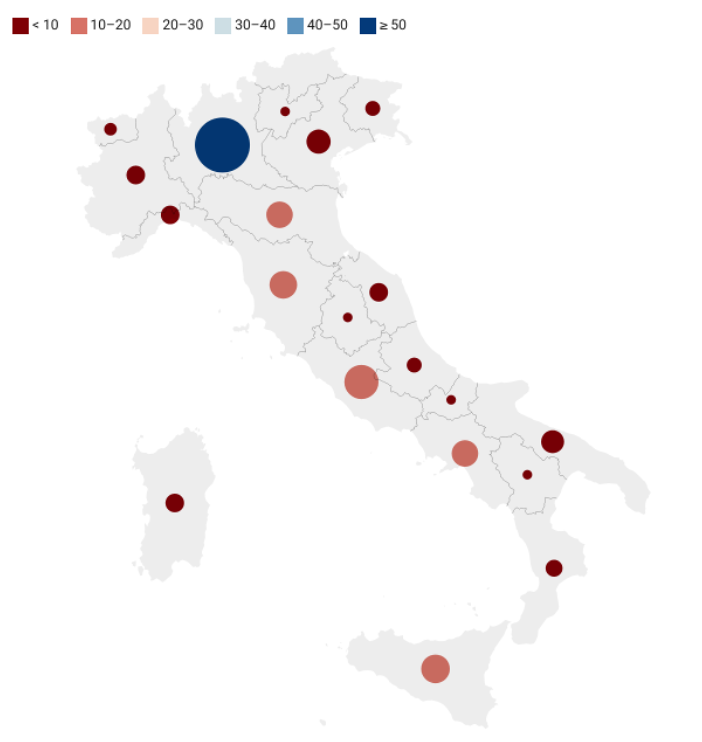


**Table S1.** Microbiological variables considered by the interviewed physicians.

|  | **Microorganism resistance pattern** | **Score** | **n (%)** |
| --- | --- | --- | --- |
| **Rectal colonisation** |  |  |  |
|  | 3GCR Enterobacteriaceae | 0 | 10 (5.8) |
|  |  | 1 | 32 (18.7) |
|  |  | 2 | 51 (29.8) |
|  |  | 3 | 78 (45.6) |
|  | CR Enterobacteriaceae | 0 | 5 (2.9) |
|  |  | 1 | 5 (2.9) |
|  |  | 2 | 26 (15.2) |
|  |  | 3 | 135 (78.9) |
|  | MBL-producing Enterobacteriaceae | 0 | 22 (12.9) |
|  |  | 1 | 12 (7.0) |
|  |  | 2 | 27 (15.8) |
|  |  | 3 | 110 (64.3) |
|  | DTR microorganisms | 0 | 8 (4.7) |
|  |  | 1 | 6 (3.5) |
|  |  | 2 | 39 (22.8) |
|  |  | 3 | 118 (69.0) |
|  | Resistant Acinetobacter baumanniii | 0 | 38 (22.2) |
|  |  | 1 | 29 (17.0) |
|  |  | 2 | 31 (18.1) |
|  |  | 3 | 73 (42.7) |
| **More than 1 site colonised** |  |  |  |
|  | CR *Acinetobacter baumanniii* | 0 | 38 (22.2) |
|  |  | 1 | 28 (16.4) |
|  |  | 2 | 32 (18.7) |
|  |  | 3 | 73 (42.7) |
|  | CR Enterobacteriaceae | 0 | 1 (0.6) |
|  |  | 1 | 11 (6.4) |
|  |  | 2 | 33 (19.3) |
|  |  | 3 | 126 (73.7) |
| **Infection** |  |  |  |
|  | CR Enterobacteriaceae | 0 | 1 (0.6) |
|  |  | 1 | 6 (3.5) |
|  |  | 2 | 28 (16.4) |
|  |  | 3 | 136 (79.5) |
|  | AmpC β-lactamase-producing Enterobacteriaceae | 0 | 9 (5.3) |
|  |  | 1 | 22 (12.9) |
|  |  | 2 | 49 (28.7) |
|  |  | 3 | 91 (53.2) |
|  | MBL-producing Enterobacteriaceae | 0 | 20 (11.7) |
|  |  | 1 | 12 (7.0) |
|  |  | 2 | 32 (18.7) |
|  |  | 3 | 107 (62.6) |
|  | DTR microorganisms | 0 | 5 (2.9) |
|  |  | 1 | 5 (2.9) |
|  |  | 2 | 41 (24.0) |
|  |  | 3 | 120 (70.2) |
|  | CR *Acinetobacter baumannii* | 0 | 30 (17.5) |
|  |  | 1 | 23 (13.5) |
|  |  | 2 | 27 (15.8) |
|  |  | 3 | 91 (53.2) |

*Note*: 3GCR, third-generation cephalosporin-resistant; CR, carbapenem-resistant; MBL, metallo-β-lactamase-producing; DTR, difficult-to-treat resistant
