# Supplementary material for: Carbapenem or new β-lactam-β-lactamase inhibitors? An Italian survey supported by SITA, SIMIT and SIAARTI to identify the factors affecting empiric antimicrobial therapy choice in real-life clinical practice
Source: Eur J Clin Microbiol Infect Dis. 2024 Mar 8;43(5):1017–23. doi: 10.1007/s10096-024-04798-8 (PMC11108948; doi:10.1007/s10096-024-04798-8)
Supplement: Supplementary file 2 — Supplementary Material 2 [file 10096_2024_4798_MOESM2_ESM.pdf]

The new versus the old: an Italian survey supported by SIMIT, SITA and SIAARTI to identify the principles of antibiotic sparing in clinical practice

Section 1: Clinicians' and Centres characteristics

* Indica una domanda obbligatoria

1. I understand no identifying information is being collected. I provide permission for information collected in this survey to be used for research purposes. By clicking yes you agree to the terms mentioned above *

Contrassegna solo un ovale.

- Yes

2. Gender *

Contrassegna solo un ovale.

- Male
- Female

3. How old are you? *

Contrassegna solo un ovale.

- < 30 years
- 30-40 years
- 40-50 years
- > 50 years

4. Which is your current position? *

Contrassegna solo un ovale.

- Trainee
- Specialist

5. Are you working in? *

Contrassegna solo un ovale.

- Infectious Diseases Unit
- Critical care medicine Unit

6. How long have you been working as a specialist? *

Contrassegna solo un ovale.

- <5 years
- 5-10 years
- > 10 years

7. Where do you work? (Please indicate the name of your Hospital) *

8. Where do you work? (Please indicate the name of your City and Region) *

9. For ID specialists/trainee only: do your daily activities include specialist consultations in other wards?

Contrassegna solo un ovale.

- Yes
- No

10. For ID specialists /trainee only: which wards does your specialist consultation activity involve?

Contrassegna solo un ovale.

- ICU
- Emergency room
- General medicine wards
- Surgery wards
- Haematology wards
- Solid organ transplant recipients wards
- All the aforementioned

Section 2: Clinical scenario and variables exploring the clinicians' propensity to start empirical antibiotic therapies

Preliminary Question

11. In daily clinical practice, would you consider empirically starting a new beta-lactams and beta-lactamase inhibitors (*Ceftazidime/avibactam, Ceftolozane/tazobactam)?* *

Contrassegna solo un ovale.

- Yes
- No

Clinical Scenario: Febrile patient; blood cultures have been collected but results are still pending.

For each of the following variables **provide the relevance that you assign in deciding if starting a new beta-lactams and beta-lactamase inhibitors** (*Ceftazidime/avibactam, Caftolozane/tazobactam)* (**0**: not relevant, **1**: low relevant; **2**: moderate relevant; **3**: extremely relevant).

12. Age > 65 years *

Contrassegna solo un ovale.

- 0
- 1
- 2
- 3
- Altro: ____________

13. Male gender *

Contrassegna solo un ovale.

- 0
- 1
- 2
- 3

14. Race (non-caucasian race) *

Contrassegna solo un ovale.

- 0
- 1
- 2
- 3

15. High BMI > 30 Kg/m2 *

Contrassegna solo un ovale.

- 0
- 1
- 2
- 3

16. Transfer from a health care facility *

Contrassegna solo un ovale.

- 0
- 1
- 2
- 3

17. For ID specialists /trainee only.
Ward of admission: Medical

Contrassegna solo un ovale.

- 0
- 1
- 2
- 3

18. For ID specialists /trainee only.
Ward of admission: Surgical

Contrassegna solo un ovale.

- 0
- 1
- 2
- 3

19. For ID specialists /trainee only.
Ward of admission: ICU

Contrassegna solo un ovale.

- 0
- 1
- 2
- 3

20. For critical care specialists /trainee only.
Tranfer from Medical ward

Contrassegna solo un ovale.

- 0
- 1
- 2
- 3

21. For critical care specialists /trainee only.
Tranfer from Surgical ward

Contrassegna solo un ovale.

- 0
- 1
- 2
- 3

22. For critical care specialists /trainee only.
Tranfer from other ICUs

Contrassegna solo un ovale.

- 0
- 1
- 2
- 3

23. Charlson Comorbidity Index *

Contrassegna solo un ovale.

- 0
- 1
- 2
- 3

24. Chronic Kidney Disease *

Contrassegna solo un ovale.

- 0
- 1
- 2
- 3

25. Diabetes Mellitus *

Contrassegna solo un ovale.

- 0
- 1
- 2
- 3

26. Immunosuppression *

Defined as: corticosteroid therapy, haematological malignancy, HIV infection, immunosuppressive therapy in autoimmune diseases

Contrassegna solo un ovale.

- 0
- 1
- 2
- 3

27. Severe neutropenia *
Defined as an absolute neutrophil count of *< 500/mmc*

Contrassegna solo un ovale.

- 0
- 1
- 2
- 3

28. Solid tumor *
chemotherapy/radiotherapy

Contrassegna solo un ovale.

- 0
- 1
- 2
- 3

29. Solid organ transplantation *

Contrassegna solo un ovale.

- 0
- 1
- 2
- 3

30. Haematopoietic stem cells transplantation *

Contrassegna solo un ovale.

- 0
- 1
- 2
- 3

31. Previous antibiotic exposure (< 90 days) *

Contrassegna solo un ovale.

- 0
- 1
- 2
- 3

32. Presence of a central venous catheter *

Contrassegna solo un ovale.

- 0
- 1
- 2
- 3

33. Mechanical ventilation *

Contrassegna solo un ovale.

- 0
- 1
- 2
- 3

34. Parenteral nutrition *

Contrassegna solo un ovale.

- 0
- 1
- 2
- 3

35. Renal replacement therapy *

Contrassegna solo un ovale.

- 0
- 1
- 2
- 3

36. Abdominal invasive procedures *

Contrassegna solo un ovale.

- 0
- 1
- 2
- 3

37. Rectal colonization by third-generation cephalosporin-resistant microorganism *

Contrassegna solo un ovale.

- 0
- 1
- 2
- 3

38. Rectal colonization by Carbapenem-resistant Enterobacteriaceae *

Contrassegna solo un ovale.

- 0
- 1
- 2
- 3

39. Rectal colonization by Metallo-β-lactamase-producing microorganism *

Contrassegna solo un ovale.

- 0
- 1
- 2
- 3

40. Rectal colonization by DTR, Difficult-to-treat resistant microorganism *

Contrassegna solo un ovale.

- 0
- 1
- 2
- 3

41. Rectal colonization by resistant *Acinetobacter baumannii* *

Contrassegna solo un ovale.

- 0
- 1
- 2
- 3

42. More than one site besides rectal colonized by resistant *Acinetobacter baumannii* *

Contrassegna solo un ovale.

- 0
- 1
- 2
- 3

43. More than one site besides rectal colonized by Carbapenem-resistant Enterobacteriaceae *

Contrassegna solo un ovale.

- 0
- 1
- 2
- 3

44. Known previous infection (< 90 days) by third-generation cephalosporin-resistant microorganism *

Contrassegna solo un ovale.

- 0
- 1
- 2
- 3

45. Known previous infection (< 90 days) by Carbapenem-resistant Enterobacteriaceae, *

Contrassegna solo un ovale.

- 0
- 1
- 2
- 3

46. Known previous infection (< 90 days) by AmpC β-lactamase-producing Enterobacteriaceae *

Contrassegna solo un ovale.

- 0
- 1
- 2
- 3

47. Known previous infection (< 90 days) by Metallo-β-lactamase-producing microorganisms *

Contrassegna solo un ovale.

- 0
- 1
- 2
- 3

48. Known previous infection (< 90 days) by Difficult-to-treat resistant microorganisms *

Contrassegna solo un ovale.

- 0
- 1
- 2
- 3

49. Known previous infection (< 90 days) by Carbapenem-resistant *Acinetobacter baumannii* *

Contrassegna solo un ovale.

- 0
- 1
- 2
- 3

50. Awareness of the resistance mechanisms epidemiology in your Centre *

Contrassegna solo un ovale.

- 0
- 1
- 2
- 3

51. Source of infection: *
Respiratory tract

Contrassegna solo un ovale.

- 0
- 1
- 2
- 3

52. Source of infection: *
Intra-abdominal

Contrassegna solo un ovale.

- 0
- 1
- 2
- 3

53. Source of infection: *
Urinary tract

Contrassegna solo un ovale.

- 0
- 1
- 2
- 3

54. Source of infection: *
Skin and soft tissue

Contrassegna solo un ovale.

- 0
- 1
- 2
- 3

55. Source of infection: *
Osteoarticular

Contrassegna solo un ovale.

- 0
- 1
- 2
- 3

56. Source of infection: *
Primary bactaeremia

Contrassegna solo un ovale.

- 0
- 1
- 2
- 3

57. Source of infection: *
Central nervous system

Contrassegna solo un ovale.

- 0
- 1
- 2
- 3

58. Presence of sepsis/sepsis shock *
Defined by Sepsis- 3

Contrassegna solo un ovale.

- 0
- 1
- 2
- 3

59. Pitt Score *
Based on:
Temperature
Mechanical ventilation
Cardiac arrest
Systolic blood pressure (SBP)
Intravenous vasopressors
Acute hypotensive episode (drop in SBP> 30 mm Hg with drop in DBP > 20 mm Hg) Mental status

Contrassegna solo un ovale.

- 0
- 1
- 2
- 3

60. Giannella Score *

Based on:
Admission to the Intensive Care Unit (ICU) Abdominal invasive procedure
Chemotherapy/radiation therapy
Number of additional colonization sites

Contrassegna solo un ovale.

- 0
- 1
- 2
- 3

61. Increment Score *

Based on:
Severe sepsis or shock at presentation
Pitt score of 6 or more
Charlson comorbidity index of
Source of BSI other than urinary or biliary tract
Inappropriate empirical therapy Inappropriate early targeted therapy

Contrassegna solo un ovale.

- 0
- 1
- 2
- 3

62. Lenght of stay < 7 days *

Contrassegna solo un ovale.

- 0
- 1
- 2
- 3

63. Lenght of stay 7-28 days *

Contrassegna solo un ovale.

- 0
- 1
- 2
- 3

64. Lenght of stay > 28 days *

Contrassegna solo un ovale.

- 0
- 1
- 2
- 3

Section 3: Exploring clinicians' personal variables influencing the choice of empirical antibiotic therapies

65. Other variables you would take into account that are not listed above

66. Briefly explain why you think it is more appropriate to spare carbapenems empirically

67. Briefly explain why you think it is more appropriate to spare the new beta-lactams and beta-lactamase inhibitors empirically

Questi contenuti non sono creati né avallati da Google.

Google Moduli
